# Anti-inflammatory potential of plant-derived extracellular vesicles from *Solanum nigrum* L. integrated in gelatine-dopamine hydrogel on RAW 264.7 and MC3T3 cells

**DOI:** 10.5599/admet.3149

**Published:** 2026-03-06

**Authors:** Anggraini Barlian, Tasya Fediarisa, Aida Fitri Kamila, Noviana Vanawati, Yung-Hsin Cheng

**Affiliations:** 1 Department of Biotechnology, Bandung Institute of Technology, Bandung, West Java, Indonesia; 2 Department of Biology, Bandung Institute of Technology, Bandung, West Java, Indonesia; 3 Scientific Imaging Center, Institut Teknologi Bandung, Bandung 40132, Indonesia; 4 Department of Materials Science and Engineering, National Taiwan University of Science and Technology, Taipei, Taiwan

**Keywords:** Cell-free therapy, lyophilization, macrophage cells, osteoblast cells, enzyme-linked immunosorbent assay

## Abstract

**Background and purpose:**

Plant-derived extracellular vesicles (PDEV) from *Solanum nigrum* L. fruit show promise as a cell-free regenerative and inflammatory therapy for bone defects due to their anti-inflammatory properties. However, challenges such as storage stability and targeted delivery efficiency remain in PDEV's applications. Strategies such as lyophilization and injectable hydrogel delivery systems offer potential solutions.

**Experimental approach:**

In this study, lyophilized PDEVs derived from *Solanum nigrum* L. berries were incorporated into a thermosensitive injectable gelatine-dopamine (Gel-Dop) hydrogel and evaluated by *in vitro* for their anti-inflammatory potential using MC3T3 pre-osteoblast cells and RAW 264.7 macrophage cells.

**Key results:**

The isolated PDEVs show a spherical morphology, an average size of approximately 132.6 nm, a polydispersity index of 0.197, and a protein concentration of 509 μg mL^-1^. These PDEVs were efficiently internalized by MC3T3 and RAW 264.7 cells after 12 hours of incubation and showed no cytotoxic effects at concentrations up to 10 μg mL^-1^. The release profile confirmed that the hydrogel effectively released the PDEVs, which remained non-toxic and were internalized by cells after 12 hours of incubation. Subsequently, treatment of lipopolysaccharide (LPS) stimulated MC3T3 and RAW 264.7 cells with PDEVs led to a reduction in IL-6 protein expression.

**Conclusion:**

These findings suggest that lyophilized PDEVs from *Solanum nigrum* L. berries, when incorporated into Gel-Dop hydrogel, hold promise for future development as an anti-inflammatory agent in bone therapy. This study is the first to characterize and incorporate lyophilized PDEVs from *Solanum nigrum* L. into thermosensitive injectable Gel-Dop hydrogel and demonstrate their anti-inflammatory potential through the suppression IL-6 expression in LPS-stimulated MC3T3 and RAW 264.7 cells.

## Introduction

Bone is the primary structure that forms the human skeleton, maintaining motor and hematopoietic function, and protecting the internal organs and nervous system [1]. Bone defects are complex clinical conditions, commonly caused by traumatic injury, osteoporotic fractures, deformities, inflammation, or tumours [2]. Globally, approximately 4 million people require bone transplants or bone replacement surgery each year [3]. Inflammation is the body’s response to infection or tissue damage, involving the activation of the immune system by cytokines such as interferons, interleukins, and chemokines. These inflammatory factors directly influence the differentiation and function of various cell types, including osteoblasts, osteoclasts, and osteocytes [4]. Chronic inflammatory diseases and certain immunosuppressive medications, such as glucocorticoids, further exacerbate bone deterioration [5].

Bone remodelling is the process of bone renewal to maintain strength and mineral balance, in which old bone is resorbed and replaced by new, mineralized matrix [6]. The process consists of osteoclast-mediated bone resorption followed by osteoblast-mediated bone formation within the bone remodelling compartment (BRC) [7]. Increasing evidence suggests that macrophages, key components of the innate immune system, influence osteoclasts and osteoblasts, modulating bone remodelling and supporting skeletal homeostasis [8]. Under inflammatory conditions, such as rheumatoid arthritis (RA), the rates of bone resorption and formation become imbalanced, leading to progressive bone loss and an increased risk of fracture, contributing to significant disease morbidity [9]. Traditional anti-resorptive therapies targeting osteoclasts effectively block bone resorption; however, because both osteoclasts and bone resorption are involved in activating the next cycle of bone formation, eliminating osteoclasts leads to a concomitant decrease in new bone formation. These anti-resorptive therapies also do not block the immune-mediated pathology that drives inflammatory bone loss [10-12].

Bone autotransplantation remains the gold standard for treating large bone defects [13]. However, this approach has several limitations, including limited donor tissue availability, donor-site morbidity, and the risk of complications such as infection and hematoma. Therefore, new, more effective and less risky therapeutic alternatives are needed [14]. Current bone engineering strategies generally utilize a combination of biomaterials and cells to create a biocompatible support structure with adequate mechanical strength. However, cell-based approaches face several challenges, including potential immune rejection, high cost, and ethical concerns. Alternatively, cell-free therapies have emerged as an innovative approach to bone tissue regeneration because they can overcome many limitations of cell-based therapies [15]. EVs are secreted by both prokaryotic and eukaryotic cells and are typically classified into ectosomes and exosomes. In regenerative medicine, exosomes have been studied as promising cell-free therapeutic candidates due to their ability to specifically target cells, low safety risks, and lower production costs. Furthermore, exosomes have shown therapeutic potential in the treatment of inflammatory diseases due to their immunomodulatory and anti-inflammatory properties [16].

Recently, plant-derived extracellular vesicles (PDEVs) have drawn growing attention as alternatives to mammalian exosomes. PDEVs share similar size, morphology, and biological activity but offer significant advantages, including low immunogenicity, abundant plant sources, and lower production costs [17]. One potential PDEV source is *Solanum nigrum* L., a plant traditionally consumed and known for its pharmacological properties, including anti-inflammatory effects [18]. Emmanuela *et al.* [19] previously explored the anti-inflammatory potential of PDEVs from *Solanum nigrum* L. fruits, suggesting their high therapeutic value in regenerative and inflammatory conditions.

Despite their promise, EVs face challenges, including storage instability. EVs stored in liquid form at -80 °C are prone to repeated freeze-thaw cycles, which can compromise biological activity [20]. In the pharmaceutical industry, solid formulations are preferred due to better long-term stability. Lyophilization is the most common approach for producing solid, easy-to-handle EV formulations. However, there are still limited published studies on EV lyophilization [21]. Furthermore, inefficient targeted delivery remains a key limitation in EV therapy, as systemically administered EVs are rapidly cleared from circulation, demanding frequent dosing. One potential solution is using hydrogels for localized delivery. Hydrogels can prolong EV retention and protect them from degradation at the injury site [22]. Particularly, injectable hydrogels that transition from liquid to gel in situ are ideal for bone regeneration due to their ability to precisely fill bone defects [23].

In this study, lyophilized PDEVs from *Solanum nigrum* L. were incorporated into a gelatine-dopamine-based injectable hydrogel and tested *in vitro* on MC3T3 osteoblast precursor cells and RAW 264.7 macrophage cell to evaluate their anti-inflammatory potential.

## Experimental

### Materials

In this study, the fruit of *Solanum nigrum* L. was purchased from the local market in Bandung, Indonesia, and the gelatine-dopamine hydrogel was provided by the Department of Materials Science and Engineering at National Taiwan University of Science and Technology.

Other materials used in this study were Dulbecco’s modified eagle medium (DMEM) - high glucose, foetal bovine serum (FBS), Antibiotic-Antimycotic (ABAM), polyethylene glycol (PEG) 6000, trehalose (Sigma-Aldrich; Merck), phosphate-buffered saline (PBS), sodium periodate (NaIO_4_) (Sigma-Aldrich), bovine serum albumin (BSA), 3-(4,5-dimethylthiazol-2-yl)-2,5-diphenyltetrazolium Bromide (MTT), 4′,6-diamidino-2-phenylindole (DAPI), dimethyl sulfoxide (DMSO), paraformaldehyde (PFA), kit BCA Assay (Pierce™ BCA Protein Assay Kit, Thermo Scientific), PKH67 Green Fluorescent Cell Linker Kits (Sigma-Aldrich; Merck), and Mouse Interleukin-6 (IL-6) ELISA Kit (BT Lab, Cat. No. E0049Mo).

### Cell culture

The MC3T3 cell line with passage 40 was maintained in *α*-MEM (Alpha Modified Eagle’s Media) culture medium, supplemented with 10 % FBS and 1 % ABAM. The RAW 264.7 cell line with passage 17 was maintained in a Dulbecco’s modified Eagle medium (DMEM) - high glucose, supplemented with 10 % FBS and 1 % ABAM. Cells were incubated at 37 °C, 95 % humidity, and 5 % CO_2_. The culture medium was changed every two to three days.

### Isolation of plant-derived extracellular vesicles from Solanum Nigrum L. berries

Fresh *Solanum nigrum* L. berries were purchased from a local market in Bandung, Indonesia. PDEV were isolated according to the method of Emmanuela *et al.* [19]. First, 250 grams of fresh fruit were washed and ground in a food grinder to produce juice. After that, the juice was filtered through 100- and 40-μm nylon filters sequentially. The filtered juice was centrifuged at 2,000*g* for 10 minutes, 6,000*g* for 20 minutes and 10,000*g* for 40 minutes. The supernatant from the last centrifugation step was mixed with polyethylene glycol (PEG) 6000 to a final concentration of 5 % (w/v). Then, the solution was stored overnight at 4 °C to help precipitate PDEV. Then, the supernatant was centrifuged at 8,000*g* for 30 minutes. PDEV was present in the pellet (precipitate) that formed. Next, the pellet was redissolved in 10 mL of bidestilled water and then filtered sequentially through 0.45- and 0.22-μm PES syringe filters. The filtered PDEV solution was then added with trehalose until it reached a final concentration of 50 mM, and then the solution was lyophilized (freeze-dried) to obtain a dry and more stable form. The results were used for experiments in this study.

### Characterization of plant-derived extracellular vesicles

PDEV morphology was characterized using a transmission electron microscope (TEM) (HT7700, 120 kV). Using the negative staining method. Dynamic light scattering (DLS) analysis was performed using a particle size analyser (Horiba SZ-100i) to measure the zeta potential of PDEV. Total PDEV protein concentration was measured using the Pierce™ BCA Protein Assay Kit (Thermo Fisher) according to the manufacturer's protocol.

### Gelatin-dopamine hydrogel containing plant-derived extracellular vesicles

Dopamine (62-31-7, Thermo Scientific) was conjugated to gelatin (type A, G2500, Sigma) in the presence of *N*-(3-Dimethylaminopropyl)-*N*’-ethylcarbodiimide hydrochloride (EDC, 25952-53-8, Aladdin) and *N*-hydroxysuccinimide (NHS, 6066-82-6, Thermo Scientific). Briefly, 1.0 g of gelatine was dissolved in 71 mL of degassed MES buffer (50 mM, pH 4.5) at 37 °C. Subsequently, 427.1 mg of NHS and 500 mg of EDC were added to the solution. After stirring for 20 minutes, 3 mL of MES buffer (50 mM, pH 3.3) was used to dissolve 901.42 mg of dopamine, which was then added to the reaction mixture. The resulting mixture was incubated at 37 °C in the dark with shaking at 100 to 150 rpm for 24 hours. The samples were dialyzed four times against acidified deionized water (pH ~3), then dialyzed against deionized water (pH ~5) for three hours. The final dopamine-grafted gelatine (Gel-Dop) product was freeze-dried and stored at -20 °C for further use. Gel-Dop solution (1 wt.%) was prepared by dissolving in PBS. Gel-Dop hydrogels were prepared by adding sodium periodate as an oxidation reagent. Sodium periodate (NaIO_4_, 7790-28-5, Macklin) was added to Gel-Dop solution to reach the final concentration of 0.125 wt.%. This study prepared four types of hydrogels: a control (no PDEV) and the other three with PDEV at concentrations of 2.5, 5, and 10 μg mL^-1^.

### Plant-derived extracellular vesicles release profile

PDEV release profile measurements were performed to determine the quantity and rate of the PDEV that was released from Gel-Dop hydrogels under *in vitro* incubation conditions. PDEV with concentrations of 2.5, 5 and 10 μg mL^-1^ was added during hydrogel preparation and incubated at 37 °C in PBS. The resulting eluate (the hydrogel’s release in PBS) was taken after 1, 3, 6, 12, 24, 48 and 72 hours, then analysed for PDEV content using the BCA test using the Pierce BCA Protein Assay Kit (Thermo Fisher).

### Intracellular uptake of plant-derived extracellular vesicles

To evaluate the uptake of PDEV into MC3T3 and RAW 264.7 cells, PDEV released from the hydrogel after 24 hours was first fluorescently labelled using the PKH67 Green Fluorescent Cell Linker kit (Merck) according to the manufacturer's protocol. The PDEV sample was mixed with diluent C and 1 μL of PKH67 was added. This mixture was incubated at room temperature for 4 minutes. Afterward, it was centrifuged at 13,000 rpm at 4 °C for 1 hour. The resulting pellet was washed with PBS and then centrifuged again at 13,000 rpm at 4 °C for 1 hour. The pellet was then dissolved in medium containing a 1 % antibiotic-antimycotic solution.

To observe PDEV internalization, 50,000 MC3T3 and 200,000 RAW 264.7 cells were grown on a confocal dish for 24 hours. After that, the culture medium was removed, and the cells were washed three times with PBS. The cells were then incubated in a previously prepared PDEV-containing medium at 37 °C in 5 % CO_2_ for 12 hours. After incubation, the cells were fixed with 4 % paraformaldehyde (PFA) for 15 minutes. Next, the cells were washed with PBS, and the nuclei were stained with DAPI (4',6-diamidino-2-phenylindole). The results of PDEV intracellular uptake into RAW 264.7 and MC3T3 cells were observed using Fluoview FV3000 Confocal Laser Scanning Microscope (Evident Corporation, Tokyo, Japan).

### Cell cytotoxicity assay

The MTT assay was employed to measure cytotoxicity in MC3T3 and RAW 264.7 cells. Cells were seeded into 96-well plates at a concentration of 10,000 cells/well in 200 μL culture medium along with varying concentrations of PDEV (1, 2.5, 5, 10 and 15 μg ml^-1^ for RAW 264.7 cells and 2.5, 5, 10, 20, and 30 μg ml^-1^ for MC3T3 cells) for two days at 37 °C and 5 % CO_2_. Then, 200 μL of the MTT solution was added to each well and incubated at 37 °C for 4 hours. The medium was then removed, and 200 μL of DMSO was added. Finally, the absorbance was measured using a Microplate reader [18].

### Enzyme-linked immunosorbent assay for interleukin-6

To test PDEV's ability to suppress the inflammatory response, an enzyme-linked immunosorbent assay (ELISA) was performed to measure interleukin-6 (IL-6) levels secreted by the cells. MC3T3 and RAW 264.7 cells were grown in 24-well plates at a cell density of 50,000 and 38,000 cells *per* well, respectively, for 24 hours. After that, the cells were pretreated for 24 hours with various concentrations of PDEV and with the eluate from PDEV-containing hydrogels, namely 2.5, 5 and 10 μg mL^-1^. After that, the cells were treated with lipopolysaccharide (LPS) for 6 h. The media used were α-MEM and DMEM-HG, supplemented with 5 % FBS and 1 % antibiotic-antimycotic. In this experiment, an anti-inflammatory negative control was used in the form of a medium with the addition of LPS and an anti-inflammatory positive control in the form of the addition of LPS and dexamethasone. IL-6 concentration was measured using a mouse IL-6 ELISA kit (BT Lab) according to the kit protocol. Absorbance was then measured at 450 nm using an iMark microplate reader (Bio Rad).

### Statistical analysis

The results are reported as mean ± standard deviation (SD), *n* ≥ 3. The data were analysed using one-way ANOVA, with a p-value < 0.05 considered statistically significant. Statistical analysis was performed using GraphPad Prism software (GraphPad Software, Inc.).

## Results

### Isolation and characterization of plant-derived extracellular vesicles

**Figure 1.**
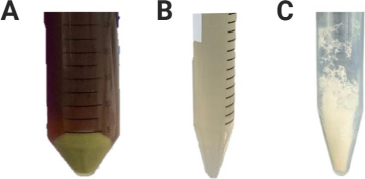
Isolation results of extracellular vesicles derived from *Solanum nigrum* L. berries; (a) PDEV precipitate, (b) PDEV solution, (c) lyophilized PDEV

PDEV from *Solanum nigrum* L. berries was successfully isolated by differential centrifugation and polyethylene glycol (PEG) precipitation. The isolation results showed a green precipitate, which is a visual indication that the nanoparticles were successfully collected (Figure 1).

**Figure 2.**
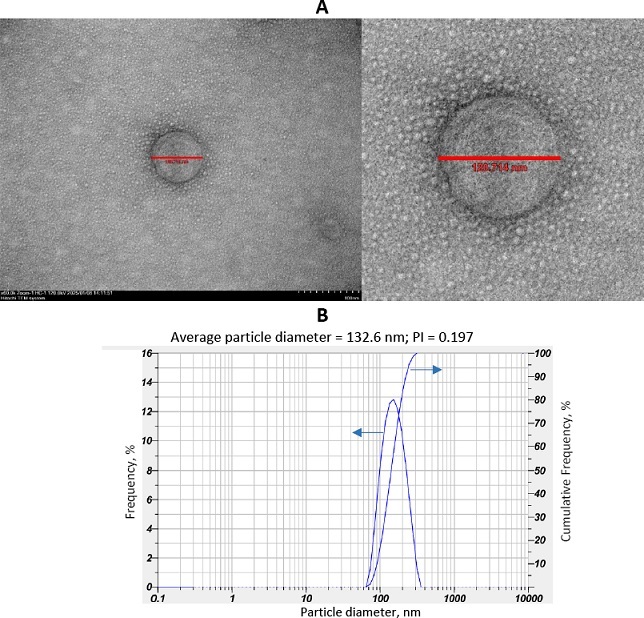
Characterization of plant-derived extracellular vesicles from *Solanum nigrum* L. berries: (A) PDEV morphology using transmission electron microscopy and (B) PDEV size distribution using particle size analyser. The solid curve represents the frequency distribution, while the dashed curve represents the cumulative percentage.

PDEV was then lyophilized to overcome the challenges of long-term storage [21]. The morphology of PDEV observed by transmission electron microscopy showed PDEV to have a spherical or cup-shaped shape, typical of exosomes/lipid nanoparticles, with a diameter of approximately 120.71 nm (Figure 2). These results were confirmed by a particle size analyser (PSA), which measured the size and homogeneity of PDEV, showing an average PDEV size of 132.6 nm and a polydispersity index (PI) of 0.197 (Figure 2). The PDEV concentration was then measured using the BCA assay, which showed a total protein concentration of 509 μg mL^-1^.

### Plant-derived extracellular vesicles release profile from Gel-Dop

**Figure 3.**
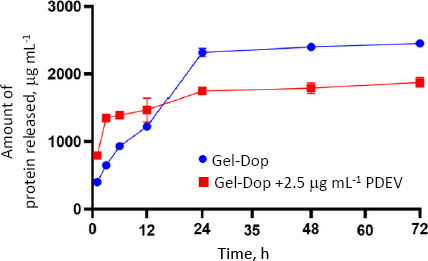
Protein release profile from Gel-Dop hydrogel and Gel-Dop hydrogel containing PDEV over 72 h. Data presented as mean ± standard deviation

After PDEV was incorporated into the gelatine-dopamine hydrogel (Gel-Dop), the hydrogel was immersed in PBS solution for 72 hours to evaluate the PDEV release profile. The eluate samples were collected at specific time intervals (1, 3, 6, 12, 24, 48 and 72 hours) and then analysed for protein content using the BCA assay as an indicator of PDEV release (Figure 3). This test was conducted to ensure that PDEV could be released gradually and effectively from the hydrogel matrix.

### Intracellular uptake of plant-derived extracellular vesicles

**Figure 4.**
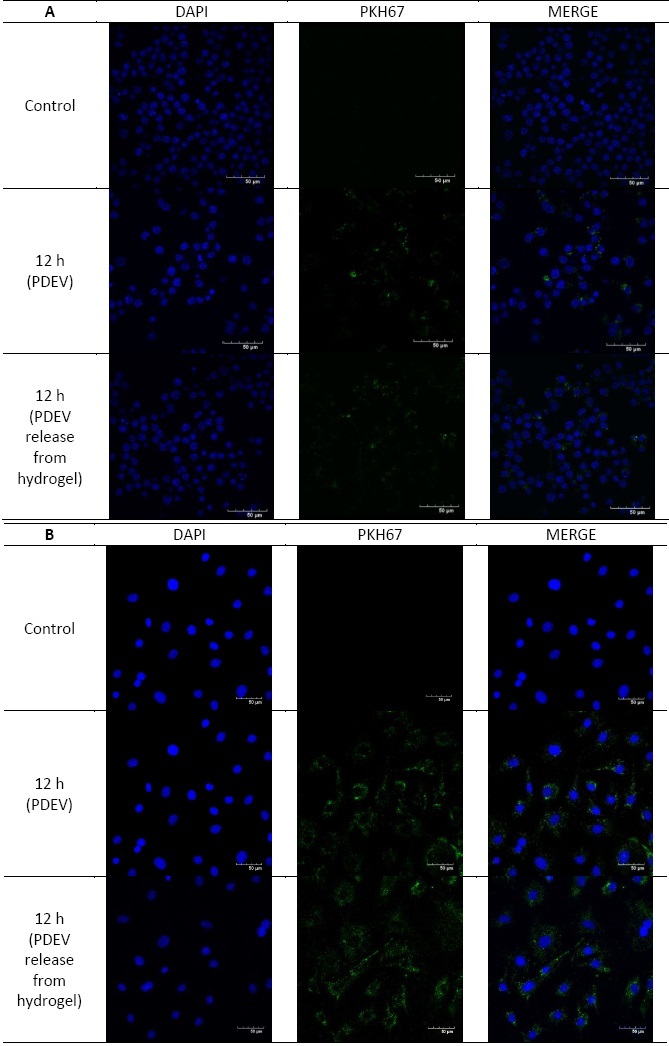
Uptake of plant-derived extracellular vesicles from *Solanum nigrum* L. berries by (A) RAW 264.7 and (B) MC3T3 cells after 12 hours of incubation using a confocal microscope. Uptake of PKH67-labeled PDEV is shown by green colour and DAPI-stained cell nucleus is shown by blue colour

Cells were incubated with PDEV labelled with PKH67, a lipophilic green fluorescent dye. PDEV uptake by RAW 264.7 and MC3T3 cells was measured at 12 hours. Cells were fixed using paraformaldehyde (PFA). Cell nuclei were stained with 4',6-diamidino-2-phenylindole (DAPI), which is shown in blue. A stronger fluorescence signal in the cytoplasm indicated greater uptake of PDEVs after incubation for 12 hours (Figure 4).

### Effect of PDEVs on cytotoxicity in MC3T3 and RAW 264.7 cells

**Figure 5.**
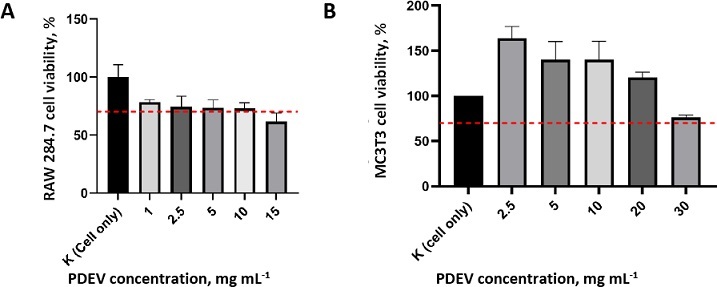
Viability of (A) RAW 264.7 cells and (B) MC3T3 cells with various PDEV concentrations. Red dashed line corresponds to the threshold for cell viability (ISO 10993-5). Data presented as mean ± standard deviation

The MTT ([3-(4,5-dimethylthiazol-2-yl)-2,5-diphenyltetrazolium bromide]) assay was used to determine the percentage of cell viability after PDEV treatment, compared with untreated cells grown in growth medium. The MTT cytotoxicity test of PDEV shows that the percentage of cell viability in each sample has a value above 70 %, indicating that each PDEV concentration up to 10 μg mL^-1^ does not have cytotoxic properties on both RAW 264.7 and MC3T3 cells (Figure 5). Then, 2.5, 5 and 10 μg mL^-1^ concentrations of PDEV were selected for incorporation into the hydrogel and continued to further testing.

**Figure 6.**
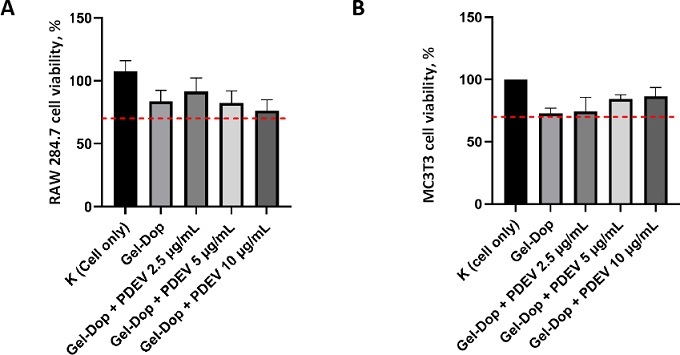
Viability of (A) RAW 264.7 dan (B) MC3T3 cells treated with Gel-Dop hydrogel and Gel-Dop hydrogel containing PDEV. Red dashed line corresponds to the threshold for cell viability (ISO 10993-5). Data presented as mean ± standard deviation

Cytotoxicity testing of Gel-Dop hydrogels incorporating PDEV was conducted to evaluate whether the hydrogels, both without PDEV and with PDEV, were biocompatible with RAW 264.7 and MC3T3 cells. Cytotoxicity testing of Gel-Dop hydrogels containing PDEV showed that both the control hydrogel (H) and the hydrogels with added PDEV (H + PDEV 2.5, 5, and 10 μg mL^-1^) were non-toxic (Figure 6). Thus, the Gel-Dop hydrogel containing PDEV can be declared safe for use and is suitable for further biological testing.

### Anti-inflammatory effect of plant-derived extracellular vesicles on LPS-stimulated RAW264.7 cells

**Figure 7.**
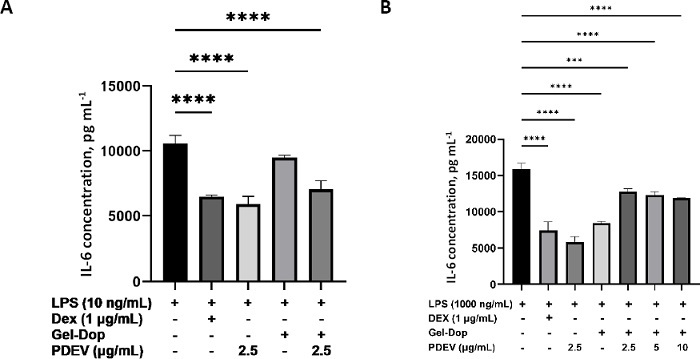
Interleukin-6 (IL-6) concentration by (A) RAW 264.7 and (B) MC3T3 cells LPS induction and PDEV (2.5, 5 and 10 μg mL^-1^) treatment released from Gel-Dop hydrogel. Data presented as mean ± standard deviation (***p < 0.001, ****p<0,0001)

In this study, IL-6 cytokine expression analysis was performed in RAW 264.7 and MC3T3 cells activated with LPS to evaluate the anti-inflammatory potential of PDEV from *Solanum nigrum* L. released from Gel-Dop hydrogel (Figure 7). The addition of PDEV alone at 2.5 μg mL^-1^ showed an anti-inflammatory effect that was even lower than that of dexamethasone, the positive anti-inflammatory control. Hydrogel treatment alone also suppressed IL-6 concentration and showed a strong anti-inflammatory effect in MC3T3 cells but not in RAW 264.7 cells. However, when PDEV (concentrations of 2.5, 5 and 10 μg mL^-1^) was incorporated into Gel-Dop hydrogel and released, the anti-inflammatory effect was not as strong as free PDEV or hydrogel alone, indicated by a higher IL-6 concentration, although it still reduced IL-6 concentration when compared to the control.

## Discussions

One of the latest studies by Emmanuela reported that PDEV from *Solanum nigrum* L. showed anti-inflammatory potential *in vitro* in RAW 267 macrophages [19]. *Solanum nigrum* L. is a plant that is widely cultivated and consumed. This plant has been shown to have various pharmacological properties, including antioxidant, antimicrobial, immunomodulatory, and anti-inflammatory effects [18]. *In vitro* and *in vivo* studies have shown that methanolic compounds and steroidal saponins extracted from *Solanum nigrum* L. berries have anti-inflammatory activity. However, there are challenges in using PDEV for regenerative therapy, including storage stability and the effectiveness of delivery to the target site.

In this study, PDEV from *Solanum nigrum* L. berries was successfully isolated using differential centrifugation and precipitation with polyethylene glycol (PEG), a precipitation agent to help precipitate nanoparticles from plant extract solution so that the nanoparticles can be collected more quickly, cheaply, and efficiently, without damaging their structure or function [24]. The isolation result showed a green precipitate, indicating that nanoparticles were successfully collected (Figure 1). Then, PDEV lyophilization was carried out to address the challenge of PDEV storage stability, as it is unstable in liquid form at -80 °C, particularly due to freeze-thaw damage that can reduce its biological activity. The PDEV solution obtained from the isolation was added with trehalose. Trehalose is a natural non-reducing disaccharide used as a preservative or cryo-preservative for easily damaged protein-based drugs, liposomes, or cells and organs. Trehalose can stabilize proteins, cell membranes, and liposomes, and reduce ice formation during freezing and prevent protein aggregation. In the exosome field, trehalose has been shown to reduce exosome fusion and reduce exosome loss during freeze-drying [25]. PDEV was then lyophilized (freeze-dried) to address its long-term storage challenge (Figure 1) [21].

This study shows that lyophilization effectively prevented PDEV aggregation, resulting in smaller, more uniform particles (lower polydispersity index) compared to fresh PDEV. Transmission electron microscopy confirmed that this process maintained the spherical and cup-shaped nanoparticle structure, consistent with previous studies. The final concentration of lyophilized PDEV was measured at 509 μg mL^-1^, though this can vary due to factors such as fruit mass and quality during isolation and PDEV stability during storage [26].

Another challenge is the limited use of PDEV due to difficulty achieving sustained slow release at specific target locations, which is hindered by body fluid flow and rapid clearance by the circulatory system. With advances in tissue engineering and biomaterials, researchers have found that combining EVs with biomaterials can overcome EV limitations in certain tissue repair applications [27]. One of the widely used biomaterials is hydrogel, which plays an important role in tissue repair and reconstruction [22]. The use of hydrogels loaded with EVs can enhance EV stability and help deliver them to the site of damage for sustained local release (in situ). Many studies have shown that EV-loaded hydrogels have great potential for tissue repair and regeneration. These can be used for almost all types of tissue damage, including skin, bone and cartilage, heart, nervous system, and reproductive organs [28].

In this study, an injectable thermosensitive hydrogel made of gelatine and dopamine was used. Injectable hydrogel is a biomaterial that offers the advantage of a minimally invasive procedure and has been widely used for bone tissue regeneration. This hydrogel can be formulated for controlled drug release [23]. Gelatine is a form of collagen that has undergone hydrolysis and denaturation, usually obtained from animal skin and bones (such as pig skin) through acid or base treatment, and then processed thermally [29]. Gelatine has the amino acid sequence Gly-X-Y and an RGD (Arg-Gly-Asp) motif that plays an important role in cell adhesion by interacting with integrins. In addition, gelatine can be naturally degraded by enzymes such as collagenase and metalloprotease, which demonstrates a biochemical affinity between gelatine and collagen [30]. As a result of partial hydrolysis of collagen, gelatine has a chemical composition that resembles the organic matrix of bone, making it a strong candidate for use as a material in bone tissue repair [31]. Dopamine can be incorporated into the hydrogel to enhance its adhesive properties and self-healing ability. The catechol group in dopamine can also interact with functional groups on the tissue surface, thereby increasing the hydrogel's tissue adhesion strength [32].

To ensure that PDEV can be gradually and effectively released from the hydrogel matrix, a PDEV release profile was conducted (Figure 3). The results showed that the Gel-Dop + PDEV group had a higher protein concentration than the control, particularly during the first 12 hours. This confirms that PDEV was successfully loaded and gradually released from the hydrogel. Interestingly, in the control group, protein release was lower overall, but it increased sharply between the 12th and 24th hours. This may be caused by the hydrogel beginning to degrade, so the increase in protein results from the release of hydrogel components due to structural degradation. The hydrogel containing PDEV did not show a similar increase in degradation, suggesting that PDEV may alter the hydrogel structure, thereby inhibiting degradation.

RAW 264.7 macrophage cells are a common model for inflammatory responses, as they are part of the innate immune system involved in inflammation regulation and pathogen phagocytosis [33-34]. In contrast, MC3T3 osteoblast cells model osteoblast differentiation and are often co-cultured with macrophages to study the impact of inflammation on this process [35-37]. Extracellular vesicles, like PDEV, can be internalized by cells, primarily macrophages, via phagocytosis [38]. This internalization is facilitated by phosphatidic acid (PA) in the PDEV membrane, known for its role in membrane fusion and fission [19,39-40]. This study demonstrates that PDEVs are taken up by both murine RAW 264.7 macrophage and MC3T3 osteoblast cell lines within twelve hours (Figure 4).

For potential therapeutic applications, PDEV should ideally exhibit no cytotoxicity and, preferably, promote cell proliferation. According to the previous study, fresh PDEV from *Solanum nigrum* L. showed no toxic effects on RAW 264.7 cells at concentrations up to 2.5 μg mL^-1^ compared with the untreated control. This current study further confirms the safety of lyophilized PDEV, with no observed toxicity for RAW 264.7 up to 10 μg mL^-1^ and for MC3T3 up to 30 μg mL^-1^ (Figure 4.). Furthermore, the eluate of PDEV-containing hydrogels at concentrations of 2.5, 5 and 10 μg mL^-1^ was also non-toxic, thus ensuring their safety for subsequent biological assays (Figure 5).

To evaluate the anti-inflammatory potential of PDEV from *Solanum nigrum* L. released from Gel-Dop hydrogel, ELISA IL-6 protein assays were performed (Figure 6). PDEV from *Solanum nigrum* L. released from Gel-Dop hydrogel was tested on MC3T3 cells induced with lipopolysaccharide (LPS) to mimic the inflammatory response. Under inflammatory conditions, IL-6 concentrations can increase and subsequently affect the activity of bone cells. Osteoblasts can be stimulated by inflammatory signals to produce IL-6. IL-6 inhibits osteoblast differentiation through activation of the JAK/STAT, SHP2/MEK2 and SHP2/AKT signalling pathways [41]. Studies have confirmed that IL-6 stimulates the expression of Receptor Activator of NF-kappa B Ligand (RANKL) in osteoblast precursors, which binds to Receptor Activator of NF-kappa B (RANK) on osteoclast precursors and promotes osteoclast cell differentiation. Therefore, in inflammatory conditions with elevated IL-6 levels, osteoblasts indirectly trigger an increase in osteoclast number and activity, leading to bone resorption [42].

Macrophages, key contributors to the inflammatory response, is involved in the secretion of IL-6 cytokines [33,43]. IL-6 production is stimulated by IL-1β and TNFα, leading to an amplified inflammatory response via M1 polarized macrophages [43]. While this M1 phenotype aids in fighting pathogens in infected tissues, M2 polarized macrophages subsequently promote anti-inflammatory responses and tissue repair [34,44]. The presence of anti-inflammatory factors is elevated in M2 macrophages, which, in turn, inhibit proinflammatory cytokine signalling pathways, thereby suppressing IL-6 secretion [43,45].

The addition of PDEV at a concentration of only 2.5 μg mL^-1^ demonstrated anti-inflammatory effects in RAW 264.7 and MC3T3 cells, even surpassing those of dexamethasone, which was used as a positive control. The anti-inflammatory potential of PDEV has been previously demonstrated by Emmanuela *et al.*, who reported that GC-MS analysis of *Solanum nigrum* L.-derived PDEV identified the presence of neral, the Z-isomer of citral, a monoterpene commonly found in lemon fruit and known for its anti-inflammatory activity [19]. In macrophages, neral inhibits NLRP3 inflammasome activation and the phosphorylation of p38 and IκB, thereby preventing the transcription of pro-inflammatory cytokine genes, including IL-6.

Treatment with the hydrogel alone also suppressed IL-6 levels and demonstrated anti-inflammatory effects, suggesting that the Gel-Dop hydrogel itself may possess intrinsic anti-inflammatory properties. A study by Zhu *et al.* [46] showed that gelatine has two main degradation products, glycine and prolyl-hydroxyproline (Pro-Hyp), which are believed to underlie the material's anti-inflammatory activity. Gelatine supplementation effectively prevented an increase in IL-6 levels in colonic tissue. Furthermore, research by Lu *et al.* [47] indicated that dopamine also has anti-inflammatory effects by inhibiting the production of the pro-inflammatory cytokine IL-6 in inflammatory conditions such as osteoarthritis. The primary mechanism behind dopamine’s ability is through inhibition of the Nuclear Factor-kappa B (NF-κB) signalling pathway. The NF-κB pathway activates many genes involved in inflammation, including those encoding cytokines such as IL-6 and inducible nitric oxide synthase (iNOS).

However, when PDEV (at concentrations of 2.5, 5, and 10 μg mL^-1^) was incorporated into the Gel-Dop hydrogel and subsequently released, its anti-inflammatory effect was not as strong as that of free PDEV or the hydrogel alone, as indicated by higher IL-6 concentrations, although still lower compared to the control. This may be due to chemical or physical interactions between PDEV and the Gel-Dop hydrogel components, which could affect the structural stability of PDEV or the bioactivity of its compounds. Therefore, further optimization of the PDEV-containing hydrogel formulation is necessary to ensure that neither the distribution nor the biological activity of the active contents in PDEV is compromised.

Additionally, the lower IL-6 concentration observed in the free PDEV treatment compared to PDEV released from the hydrogel may also be explained by differences in exposure profiles. In the free PDEV treatment, which is applied directly to the cells, the entire dose of PDEV is immediately available, resulting in rapid and strong anti-inflammatory responses. In contrast, when PDEV is embedded within the hydrogel, it is released gradually into the cellular environment, resulting in a lower concentration of active and bioavailable PDEV. Although the effect appears weaker in this *in vitro* test, the use of hydrogels is intended to address the challenge of inefficient PDEV delivery to target tissues. Therefore, this slow-release system is desirable for *in vivo* therapeutic applications, as it can provide sustained therapeutic effects at the target site over a longer period.

## Conclusions

This study is the first to characterize and incorporate lyophilized PDEVs from *Solanum nigrum* L. berries into thermosensitive injectable gelatine-dopamine (Gel-Dop) hydrogel and demonstrate their anti-inflammatory potential through the suppression ofIL-6 expression in LPS-stimulated MC3T3 and RAW 264.7 cells. The lyophilized PDEVs exhibited a spherical morphology, an average size of approximately 132.6 nm, a polydispersity index (PI) of 0.197, and a protein concentration of 509 μg mL^-1^. These PDEVs were efficiently internalized by MC3T3 and RAW.267 cells within 12 hours of incubation and showed no cytotoxic effects, maintaining cell viability above 70 % at concentrations up to 10 μg mL^-1^. Varying doses of PDEVs were then incorporated into the gelatine-dopamine hydrogel and assessed for their anti-inflammatory activity. The hydrogel effectively released the PDEVs, which remained non-toxic and were internalized by cells after 12 hours. Subsequently, treatment of LPS-stimulated MC3T3 and RAW 264.7 cells with PDEVs led to a reduction in IL-6 protein expression. These findings suggest that lyophilized PDEVs from *Solanum nigrum* L. berries, when incorporated into Gel-Dop hydrogel, hold promise for future development as an anti-inflammatory agent in bone therapy.
